# Disinhibited Eating and Executive Functioning in Children and Adolescents: A Systematic Review and Meta-Analysis

**DOI:** 10.3390/ijerph192013384

**Published:** 2022-10-17

**Authors:** Clarissa V. Shields, Kara V. Hultstrand, Caroline E. West, John J. Gunstad, Amy F. Sato

**Affiliations:** 1 Department of Psychological Sciences, Kent State University, Kent, OH 44242, USA; 2 Brain Health Research Institute, Kent State University, Kent, OH 44242, USA

**Keywords:** executive functioning, disinhibited eating, overeating, obesity

## Abstract

A growing body of research suggests disinhibited eating and weaker executive function (EF) are two risk factors for pediatric obesity. Emerging brain imaging and behavioral findings support the notion that EF skills impact eating regulation. However, a major gap in the current literature is a synthesis of the association between various EF skills and disinhibited eating patterns across child development. To address this gap, a systematic review and meta-analysis was conducted to examine the effect of EF skills on disinhibited eating behaviors among youth ages 3–18 years old. PubMed and PsychINFO databases were utilized and data from 15 studies with a total sample of 4909 youth were included. A random effects meta-analysis revealed a small negative effect of overall EF skills on disinhibited eating behavior, *r* = −0.14, *p* < 0.01. Analysis of individual EF skills found working memory had an overall medium negative effect on disinhibited eating behavior, *r* = −0.25, *p* < 0.05. Taken together, findings from this meta-analysis support an inverse relationship between EF abilities and disinhibited eating patterns in children and adolescents, such that poorer EF abilities are associated with higher levels of disinhibited eating. Given the effect on eating behavior, future research is needed to assess whether EF difficulties may be a barrier to effective weight management in youth. Specifically, research is needed to examine whether EF skills may be a key target to consider for effective obesity prevention and treatment in children and adolescents.

## 1. Introduction

A growing body of literature links risk for pediatric obesity to disinhibited eating behaviors [1] as well as deficits in cognition, particularly executive functioning (EF) [2]. This is concerning since pediatric obesity is associated with adverse physical and psychological comorbidities, including diabetes, hypertension, and depression [3]. Children and adolescents with weaker EF may have a harder time regulating their food intake, which may increase risk for obesogenic eating patterns (i.e., disinhibited eating) and higher adiposity [4,5]. As the literature surrounding neurocognitive correlates of disinhibited eating in children and adolescents continues to grow, there has yet to be a synthesis of these current findings. This present gap is notable because such findings may be relevant to the development of novel pediatric weight management interventions.

### 1.1. Disinhibited Eating and Executive Functioning

Disinhibited eating is defined as the tendency to overeat in response to internal (e.g., emotional distress) or external (e.g., palatable food) cues other than hunger and affects approximately 60% of youth [6]. Given the variety of internal and external cues that may prompt overeating, Shomaker and colleagues proposed that disinhibited eating is best conceptualized as an umbrella term that includes the different but overlapping constructs of eating in the absence of hunger (EAH), emotional eating (EE), loss of control (LOC), and binge eating (BE) [6]. EAH is the tendency to eat in response to palatable foods, typically high in energy density (e.g., cookies, chips), past satiety [7]. Similarly, EE is the tendency to eat palatable foods in the absence of hunger, but in response to emotional distress such as stress, sadness, or anxiety [8]. LOC is another type of disinhibited eating pattern defined as subjectively feeling unable to control or stop the type and amount of food eaten [9]. While LOC captures subjective feelings of disinhibited eating, BE involves overeating objectively large amounts food during a discrete period of time while experiencing a sense of loss of control [10]. It is important to understand developmental risk factors, such as weaker EF skills, for disinhibited eating patterns in youth. These risk factors may not only impact pediatric obesity risk but may potentially also have implications for guiding future research on pediatric obesity prevention and management strategies.

From a neurodevelopmental perspective, EF is one emerging individual factor that has been linked to disinhibited eating in youth. Theoretical models of eating regulation suggest that self-regulation of appetitive cues and eating behavior is dependent on EF abilities [11,12]. Executive functions are “higher-level” cognitive processes that aid in the control of goal-directed behaviors [13] and are divergent from general intelligence [14]. The ability to regulate and adjust food intake is proposed to be an innate ability that begins in infancy [15] and is then shaped by parent feeding practices [16]. While parents may influence youth’s eating environments, EF is also associated with disinhibited eating patterns in children and adolescents, particularly in situations where youth are in control of the amount of food consumed [17]. Developmentally, disinhibited eating increases with age and most commonly occurs during adolescence [6]. Adolescence is a critical time for increased autonomy [18] and maturation of brain structures that mediate EF skills [19]. Specifically, as the brain develops through childhood and adolescence, EF processes undergo both progressive (e.g., increased myelination) and regressive changes (e.g., synaptic pruning) for optimal functioning [20]. These anatomical changes suggest relations between EF and disinhibited eating may vary across development where children and adolescents are at greatest risk for difficulties with disinhibited eating.

### 1.2. Behavioral Studies Examining Executive Functioning and Disinhibited Eating

Across different forms of assessments, pediatric studies have found an association between lower EF and higher levels of various disinhibited eating patterns, including BE [21,22], LOC [23,24,25], EE [26,27,28], and EAH [29,30,31,32,33]. Several studies in youth have broadly examined EF as a unitary construct, with correlation coefficients indicating medium-to-large effects on parent-reported EE in preschoolers [26,27] and BE symptoms in adolescents [21]. Zhou and colleagues found that child effortful control, an analogous construct to EF top-down regulation [34], reported by parents was associated with objectively measured EAH in preschoolers [33]. However, researchers have also examined parent-reports of child effortful control on objective EAH in preschoolers and found no effect [26,29,35]. Among adolescents, parent-reported global EF was weaker in adolescents with LOC [27]. These mixed findings across different EF and disinhibited eating measures make it difficult to understand the overall association between EF and disinhibited eating in children and adolescents. While a recent systematic review proposed an association between child EF and eating regulation [36], to date there have been no meta-analyses quantitatively synthesizing the overall effect of EF on disinhibited eating in youth to guide future research.

### 1.3. Core EF Components and Eating Regulation

When seeking to understand EF in the context of eating regulation, it is important to note that EF is a latent construct made of separate but partially related skills [37]. One common model of EF validated and used in the child literature is the three “core” factor model, which includes: (1) inhibitory control, (2) working memory, and (3) cognitive flexibility [38]. Inhibitory control reflects the ability to stop or prevent an automatic response to achieve a short- (e.g., eating a small bowl of chips instead of a bag) or long-term goal (e.g., choosing an apple instead of a cookie for a snack). Weaker inhibitory control has been associated with greater levels of emotional eating [26] and EAH [29,30,31] in preschool- and school-aged children. However, a couple of studies found no association between inhibitory control and EE [39], EAH [39,40], or LOC [24,25] among school-aged children and adolescents.

Cognitive flexibility primarily aids in the ability to switch back and forth between different tasks or goals [41]. Cognitive flexibility may predict a youth’s ability to successfully switch between goals of regulating emotions and stopping eating to adhere to long-term dietary goals [36]. While adult studies suggest an inverse association between cognitive flexibility and disinhibited eating [42], studies examining this association in youth have found no effect [24,26,39]. Working memory involves the ability to maintain and update relevant information, such as satiation or long-term health goals [43]. Weaker working memory abilities have been associated with higher frequencies of binge eating and LOC among school-aged children and adolescents [21,24]. In contrast, among children ages 6–11, Groppe and Elsner found working memory had no association with emotional or external eating [39].

Given the mixed literature, it is important to consider variables that may contribute to the varying observed effects of EF on disinhibited eating, potentially serving as moderators of the association between these variables. Disinhibited eating increases with age and most commonly occurs during adolescence [6]. The neural changes that occur during puberty potentially make the association between EF and disinhibited eating stronger in adolescents compared to younger children. Females also tend to experience puberty earlier than males [44]. Further, several studies have found a positive association between disinhibited eating and higher adiposity in youth [1,45]. Taken together, the association between EF and disinhibited eating may be strengthened among older adolescents, females, and youth classified as overweight or obese. However, there is currently a lack of empirical research systematically examining these variables across studies.

### 1.4. Present Study

Previous research has examined associations between EF and disinhibited eating patterns, but findings are inconsistent and difficult to generalize given the various assessment methods utilized. Much of the current research also focuses on clinical adolescents and adults with an eating disorder, which is problematic since disinhibited eating also emerges in childhood and can occur at subclinical levels. To move the literature forward, this study takes a novel approach to quantitatively examine the overall association between EF and various disinhibited eating behaviors among non-clinical children and adolescents. The primary goal of the present study is to examine the effect of EF skills on disinhibited eating behaviors among youth ages 3–18 years old via a systematic review and meta-analysis. It is hypothesized that overall EF will be negatively associated with disinhibited eating behaviors among non-clinical youth. The second goal is to examine the overall effect of each EF component on disinhibited eating in non-clinical youth. It is hypothesized that inhibitory control, cognitive flexibility, and working memory will be negatively associated with disinhibited eating behaviors among non-clinical youth. Lastly, this study aims to explore potential moderators (e.g., age, biological sex, weight status) that may impact the relation between EF and disinhibited eating patterns. Synthesized findings from this novel study may help to further understand the role of EF abilities within the context of eating, thus pointing to new potential targets of intervention to reduce obesity risk and promote healthy weight management among youth.

## 2. Methods

### 2.1. Retrieval of Studies

A systematic literature search was conducted in *PsychINFO* and *PubMed* in accordance with PRISMA-Statement guidelines [46]. Searches were conducted in July 2020 and all relevant articles published up to June 2020 were evaluated for inclusion in the present meta-analysis. Search terms included “executive functioning” OR “executive control” OR “self-control” OR “cognitive control” OR “impulsivity” OR “negative urgency” OR “positive urgency” OR “inhibitory control” OR “response inhibition” OR “working memory” OR “cognitive flexibility” OR “set-shifting” AND “disinhibited eating” OR “loss of control” OR “binge eating” OR “eating in the absence of hunger” OR “emotional eating” OR “external eating” OR “uncontrolled eating” OR “stress-induced eating” OR “overeating” OR “snack intake” OR “food intake” OR “eating”. This wide array of terms ensured a comprehensive search of the literature as several different terms are used to reference EF [47] and disinhibited eating in the child and adolescent literature [6]. Literature searches were limited to articles published in English and child or adolescent age range (i.e., preschool child, school-aged child, adolescent). Dissertation abstracts were included in the search to reduce risk of publication bias.

### 2.2. Inclusion Criteria

To be eligible for inclusion, studies were required to (a) include a cross-sectional research analysis design, (b) be published in English during or before June 2020, (c) examine a sample of children and/or adolescents between the ages of 3 and 18, (d) include a computerized, experimenter-administered, parent self-report, teacher self-report, or youth self-report measure of EF that assessed one of the following: general executive functioning, inhibitory control, working memory or cognitive flexibility/set-shifting, (e) include an objective or subjective measure of disinhibited eating that assessed at least one of the following types of eating behaviors: disinhibited eating, binge eating, loss of control, emotional eating, eating in the absence of hunger, or external eating, (f) include non-treatment seeking (e.g., weight management, psychotherapy) youth.

Studies were excluded if analyses included treatment-seeking youth for weight management, an eating disorder (i.e., binge eating disorder (BED), bulimia nervosa (BN), or anorexia nervosa (AN) as defined by DSM-5 criteria, or a psychopathology disorder (e.g., depression, substance abuse). Studies were also excluded if they included youth with a diagnosed neurological disorder (e.g., attention-deficit hyperactivity disorder (ADHD), autism, fetal alcohol syndrome disorder (FASD), epilepsy, traumatic brain injury (TBI). While ADHD is primarily characterized by EF deficits, it has other neurological complexities that may confound results [48]. Studies that assessed ADHD symptoms (e.g., executive functioning) in children without a clinical ADHD diagnosis as defined by the DSM-5 were still included. Lastly, studies that assessed disinhibited eating through objective (e.g., total kCals consumed) or subjective food intake (e.g., snacking frequency) without controlling for hunger were excluded since disinhibited eating reflects eating patterns that occur in response to cues other than hunger.

**Figure 1:**
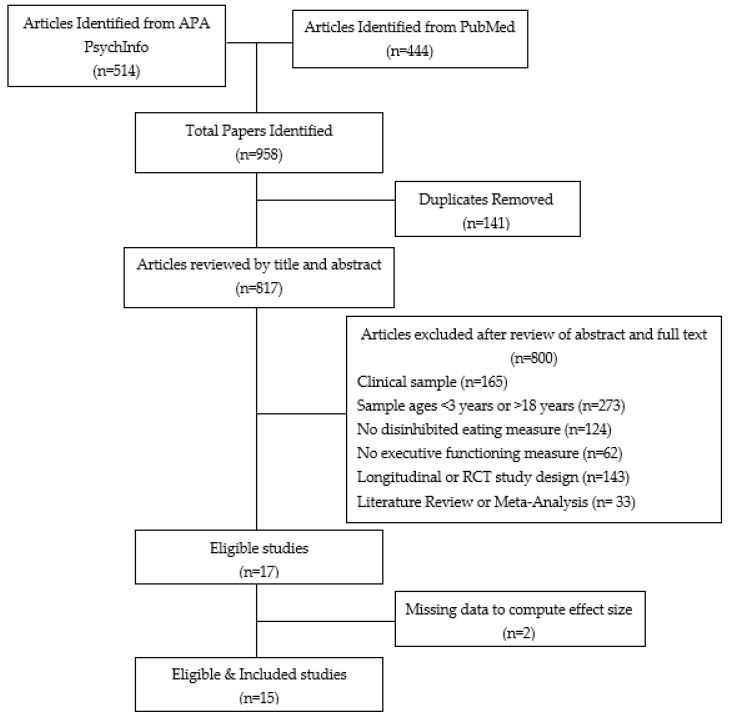
Consort flow diagram.

A total of 958 studies were identified from the literature search and screened for eligibility by two independent evaluators. A third independent evaluator reviewed discrepancies in identified eligible articles (*n* = 3) to determine final inclusion. Following review of abstracts and full text, 17 studies were determined eligible for inclusion. Two studies did not provide sufficient data to calculate the effect size of an association between EF and disinhibited eating [49,50]. The final sample included 15 eligible studies for the present meta-analysis. See Figure 1 for the PRISMA consort flow diagram.

Eligible studies were coded using a systematic coding procedure. Sample characteristics, EF and disinhibited eating constructs assessed, method of assessment (objective vs. subjective), and administered EF and disinhibited eating measure(s) were coded for each article (see Table 1). Given that a variety of different EF and disinhibited eating assessments were conducted within studies, standardized Fischer Z effect size correlations were calculated for each EF and eating assessment correlation within studies.

### 2.3. Executive Functioning Tasks

EF tasks were coded as overall EF or an individual EF component based on the 3 “core” factor model that includes inhibitory control, cognitive flexibility, and working memory [51]. Impulsivity was not included as an EF task since it is considered a separate multi-faceted construct that only partially overlaps with EF [52]. Overall assessment of EF was assessed with subjective measures such as the BRIEF Global Executive Composite (*n* = 2), BRIEF Behavioral Regulation Index (*n* = 3), CBQ Effortful Control Scale (*n* = 4), Self-Control Scale (*n* = 1), and Self-Regulation of Eating Questionnaire (*n* = 1). A composite of objective assessor administered tasks also assessed overall EF (*n* = 1). If studies included multiple assessments of EF, a standardized effect size was calculated for each EF assessment. The mean standardized effect size coefficient (ES) for each study was then included in the present meta-analysis. See Table 1.

#### 2.3.1. Inhibitory Control

Several different types of assessments assessed inhibitory control in the child and adolescent literature. This included the Stop-signal Task (*n* = 1), Go/No-Go Task (*n* = 1), Fruit Stroop Task (*n* = 1), Flanker Task (*n* = 2), Tapping Task (*n* = 1), Gift Delay Task (*n* = 1), Ability to Delay Gratification (*n* = 1), and Delay of Gratification Task (n = 4). These tasks all assessed the ability to withhold or delay an automatic or prepotent response. The BRIEF Inhibition Subscale (*n* = 2), CBQ Inhibitory Control Subscale (*n* = 1), and Desired Results Developmental Profile Impulse Control Subscale (*n* = 1) also subjectively assessed inhibitory control by parent or self-report. The BRIEF includes the Behavioral Index which contains subscales for both inhibitory control and cognitive flexibility. Since this index included two different core EF components, it was not coded for inhibitory control or cognitive flexibility to reduce risk of confounding results. A total of 11 studies examined the relation between inhibitory control and disinhibited eating in non-clinical youth.

#### 2.3.2. Working Memory

Only two tasks objectively assessed working memory in the child and adolescent literature, the List Sorting Task (*n* = 1) and Digit Span Backwards (*n* = 1). These tasks required participants to hold information for a short period of time and update it with new information. The BRIEF Metacognitive Index (*n* = 1) also subjectively assessed working memory by parent and self-report. A total of three studies examined the relation between working memory and disinhibited eating in non-clinical youth.

#### 2.3.3. Cognitive Flexibility

Tasks that assessed cognitive flexibility in the child and adolescent literature included the Dots Task (*n* = 1), Dimensional Change Card Sort (*n* = 1), and Cognitive Flexibility Task (*n* = 1). Cognitive flexibility tasks assessed the ability to provide a stimulus-response action and then switch to a different stimulus-response action. A total of three studies examined the relation between cognitive flexibility and disinhibited eating.

### 2.4. Disinhibited Eating

Disinhibited eating was assessed as EAH (*n* = 9), EE (*n* = 5), LOC (*n* = 3), and BE (*n* = 1). Regarding EAH, several different measures were used including the CEBQ Food Approach Scale (*n* = 2), CEBQ Food Responsiveness Subscale (*n* = 3), DEBQ-C External Eating Scale (*n* = 2), and EAH Task (*n* = 6). Emotional eating was assessed using the DEBQ-C Emotional Eating Scale (*n* = 1), CEBQ Emotional Overeating Subscale (*n* = 3), and Emotional Eating Scale (*n* = 1). The chEDE (*n* = 2) assessed LOC, while both the chEDE-Q (*n* = 1) and BES (*n* = 1) assessed binge eating symptoms.

### 2.5. Moderators

To assess moderators in a meta-analysis, it is recommended that each moderator has a minimum of 10 studies [53]. Therefore, age, gender, and weight status were coded as continuous moderators due to the limited numbers of studies. Mean age, percentage female, and percentage of sample classified as overweight or obese were coded for each eligible study. Only 10 studies provided information on child weight status and were included to examine weight status as a continuous moderator. Moderators were not examined for the associations between inhibitory control, working memory, or cognitive flexibility and disinhibited eating due to the small number of studies [54]. See Table 1.

## 3. Analytic Plan

Analyses were conducted in Comprehensive Meta-Analysis Software Version 3 (BIOSTAT, Englewood, NJ, USA, 2013). First, the mean effect size was computed for each study by calculating Fischer’s Z for every EF and disinhibited eating correlation in the study and then computing mean standardized effect sizes (ES) This method is preferable due to the multiple methods used to examine an effect within a study [54]. A random effects meta-analysis was then conducted to determine the overall strength of the association between EF and disinhibited eating. The 95% confidence intervals of overall effects and weighted effect were also computed. See Table 1. Second, mean standardized effect sizes were computed across measures of inhibitory control, working memory, and cognitive flexibility in each study that assessed individual EF components. Meta-analyses were also conducted for inhibitory control, working memory, and cognitive flexibility. Several methods were utilized to determine presence of publication bias and determine robustness of findings. First, a funnel plot analysis was conducted as a quantitative measure of publication bias. Second, an Egger’s regression analysis was conducted to quantitively measure publication bias. The fail-safe number (FSN) was also computed to determine the minimum number of studies with null findings needed for significant results to become non-significant. Heterogeneity tests were also conducted using Hedges’ Q test and I^2^ Index. Finally, meta-regressions examined age, biological sex, and weight status as potential moderators of the relation between EF and disinhibited eating.

## 4. Results

### 4.1. Descriptive

Eligible articles (N = 15) in the meta-analysis included a total sample of 4909 youth, with sample sizes ranging from 29 to 1657 across studies. Study samples included youth between the ages of 3 and 18, with mean ages ranging from 4.17 to 16.7 years (see Table 1). Studies had relatively equivalent numbers of males and females, with samples ranging from 47.5 to 67.2 percent female. Only 10 studies reported child weight status, with children classified as overweight or obese comprising 8.75–50% of the samples. Subjective (*n* = 6), objective performance-based (*n* = 6), or a combination of subjective and objective assessments (*n* = 3) assessed EF. Method for assessing disinhibited eating was primarily subjective (*n* = 11), with only a few studies objectively assessing EAH (*n* = 6).

### 4.2. Overall EF

**Figure 2:**
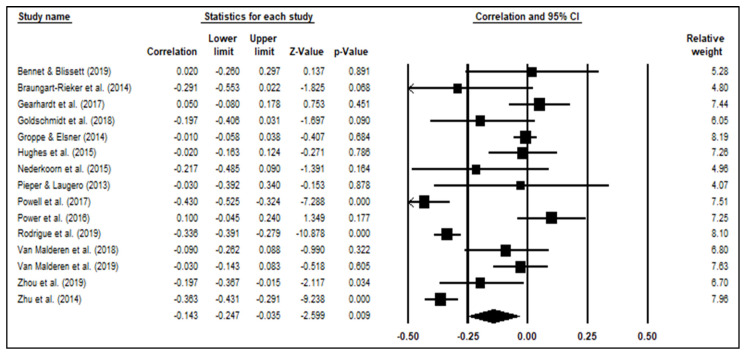
Random effects correlations, 95% confidence intervals, forest plot, and relative weight of overall executive functioning on disinhibited eating.

There was a significant small correlation between overall EF and disinhibited eating, *r* = −0.14, *p* < 0.01. Overall, poorer EF was associated with higher levels of disinhibited eating in youth. Results from the funnel plot indicate that publication bias is unlikely based on low variance between standard errors (see Figure 2). Egger’s regression was also not significant, *t*(13) = 0.06, *p* = 0.95, indicating no publication bias. Further, the FSN was 296, which indicates that findings are robust since it exceeds the criteria of 85 [55]. Hedges’ Q test was also significant, *Q*(14) = 157.84, *p* < 0.001, indicating heterogeneity and support for a random effects model. The I^2^ Index was 91.13%, which suggests a large amount of heterogeneity [56]. Age, biological sex, and weight status did not moderate the association between EF and disinhibited eating behaviors.

### 4.3. Inhibitory Control

Inhibitory control was not associated with disinhibited eating, *r* = −0.003, *p* = 0.85. Hedges’ Q test was not significant, *Q*(2) = 7.24, *p =* 0.612, indicating homogeneity of results. The I^2^ Index was 0%, also indicating homogeneity. Egger’s regression was not significant, *t*(1) = 0.63, *p* = 0.55, suggesting no publication bias. Funnel plot analyses also indicated no publication bias.

### 4.4. Working Memory

There was a significant medium correlation between working memory and disinhibited eating, *r* = −0.25, *p* < 0.05. Both Hedges’ Q test, *Q*(2) = 54.23, *p* < 0.001, and the I^2^ Index of 96.31% indicated a large amount of heterogeneity. Egger’s regression, *t*(13) = 0.06, *p* = 0.95, indicated no publication bias. However, funnel plot analyses suggest potential publication bias. The FSN was 52, which suggests findings are robust since it is above the 25 criteria for a sample of three studies [54].

### 4.5. Cognitive Flexibility

There was no overall association between cognitive flexibility and disinhibited eating, *r* = −0.018, *p* = 0.45. Hedges’ Q test was not significant, *Q*(2) = 1.48, *p =* 0.48, indicating homogeneity of results. The I^2^ Index was 0%, also indicating homogeneity. Egger’s regression, *t*(1) = 0.11, *p* = 0.93, and funnel plot analysis suggest no publication bias.

## 5. Discussion

The purpose of the present meta-analysis was to examine the overall association between EF and disinhibited eating in non-clinical children and adolescents. Results provided support for overall EF, specifically working memory, as cognitive factors that are associated with disinhibited eating in youth. Overall, EF showed a small correlation with disinhibited eating patterns across 15 different studies such that weaker EF was associated with higher levels of disinhibited eating patterns among non-clinical youth. Regarding individual EF components, working memory showed a medium inverse correlation with disinhibited eating. Inhibitory control and cognitive flexibility were not associated with youth’s disinhibited eating behaviors. Additionally, this study examined potential moderators of the relation between EF and disinhibited eating, and age, biological sex, and weight status had no interactive effects.

### 5.1. Overall Executive Functioning and Disinhibited Eating

With regard to disinhibited eating, findings from the present study are consistent with the dual-process model, which proposes hypoactive reflective processes (e.g., EF) predict overeating [57]. The model suggests overeating occurs as a result of failure to regulate cravings for rewarding cues, such as food [58]. According to the dual process model, if automatic processes are stronger than EF processes, then overeating is more likely to occur [57]. However, due to the cross-sectional nature of findings within the present study directionality cannot be inferred. Findings regarding overall EF are also consistent with another recent meta-analysis that found overall EF had a small overall correlation with child and adult health behaviors, including diet and physical activity [59]. Therefore, it is possible that examination of overall EF in children and adolescents is more robust than individual components that mature later in adolescence or early adulthood [60].

It is also important to consider that disinhibited eating, particularly among children and adolescents, may evolve from gene–environment interactions [61], with EF reflecting only one possible genetic factor associated with disinhibited eating in youth [14]. Further research is needed to understand how associations between EF and eating may be impacted by different environmental contexts. Additionally, given that young children and adolescents may not have intent to control food intake, the concept of disinhibited eating may need to be conceptualized differently throughout different stages of development. For example, disinhibited eating in youth, particularly in early childhood, may simply reflect propensity to eat high levels of food intake rather than failure of self-control, unless examined within contexts of dietary intentions. Taken together, the association between youth’s EF and disinhibited eating behaviors found in the present study reflects only one potential individual component associated with disinhibited eating in non-clinical youth.

Although overall EF had a small correlation with disinhibited eating in non-clinical youth, there was a large amount of heterogeneity among studies for which the study moderators did not account. With respect to age, our findings were in line with those of Kelly and colleagues, who found that age (children versus adolescents) did not moderate the effects of EF on total food intake during a laboratory eating buffet procedure in non-treatment seeking youth [62]. While EF abilities increase with age [60], puberty onset may also impact the association between EF and disinhibited eating in youth [63], impacting both automatic and reflective cognitive regulation processes. It is possible that puberty onset and stage of development, rather than age, may have an interactive effect on EF and disinhibited eating relations in youth.

In addition, biological sex was not observed to moderate the association between EF and disinhibited eating. One possible explanation for non-significant findings in the present study is the relatively equivalent presentation of males and females across studies. The percentage of females in the eligible articles only ranged from 47.5 to 67.2. Yet, similar to present findings, gender did not predict different levels of emotional eating in adolescents in another study [64]. A meta-analysis among adults found biological sex only moderated automatic processes such as impulsivity, but not EF [65]. Among youth, it is plausible that no biological sex differences exist between the association of EF and disinhibited eating behaviors. However, more research is needed to determine robustness of these findings.

Contrary to hypothesis, weight status also did not moderate the association between EF and disinhibited eating in youth. This seems counterintuitive given that approximately 30% of youth classified as overweight or obese report LOC and binge eating symptoms [66]. Research has primarily examined EF differences in non-clinical adolescents with or without LOC [25], neglecting how youth with obesity with and without disinhibited eating behaviors may also differ. Further, few studies in the present meta-analysis examined z-BMI [24,25,31,39] or BMI percentile [26]. Given that obesity is also associated with weaker EF [2] and increased sensitivity to food cues [67] in non-treatment seeking youth, it is possible that EF has a different relation with disinhibited eating in youth with obesity compared to non-overweight youth. Future research to still needed to untangle these mixed findings. This is particularly important with regards to weight management since weaker EF in youth predicts a poorer response to pediatric obesity treatments [68].

### 5.2. EF Components and Disinhibited Eating

#### 5.2.1. Working Memory

Findings from the present meta-analysis suggest that working memory appears to be a specific EF skill that uniquely predicts disinhibited eating in youth. Specifically, weaker working memory was associated with higher levels of disinhibited eating. Compared to other EF components, working memory may have a relatively stronger association with disinhibited eating than overall EF since it aids in processing food and satiation cues [43]. Adolescents engaging in disinhibited eating may have greater attentional biases towards food cues, which has been associated with greater reward sensitivity [69]. Working memory has also been inversely associated with food cravings in adults [70]. Therefore, a stronger working memory may also aid in decreasing sensitivity to food cues and cravings in youth. While consistent with the literature, these findings should be interpreted with caution given that only three studies examined relations between working memory and disinhibited eating, with potential publication bias. In addition, since the present study cannot infer causation due to the cross-sectional nature of findings, future research is needed to examine mechanisms through which working memory is associated with disinhibited eating in youth.

#### 5.2.2. Inhibitory Control

Inhibitory control was not associated with disinhibited eating in the present study. This is similar to findings in other studies among children and adolescents [62,71]. For instance, Lavagnino and colleagues found inhibitory control did not differ among children classified as overweight with and without BED [71]. In contrast, Kittel and colleagues found adolescents with BED and obesity had poorer inhibitory control compared to adolescents with obesity without BED [72]. It is possible that inhibitory control may not be related to disinhibited eating in youth since these neural processes do not fully mature until adulthood [73]. The complexity of inhibitory control assessment may also account for the lack of association with disinhibited eating in the present study.

#### 5.2.3. Cognitive Flexibility

Similar to the present study, other studies among adults did not find an association between cognitive flexibility and disinhibited eating [74]. Only three studies examined the effect of cognitive flexibility on disinhibited eating behaviors in youth, indicating results should be interpreted with caution given the low generalizability. It is possible that cognitive flexibility is not related to disinhibited eating behaviors in youth. Another possibility is that weaker cognitive flexibility occurs as an adverse effect of obesity rather than a pre-dispositional risk for disinhibited eating. Controlling for adiposity and eating disorder diagnoses has eliminated associations between cognitive flexibility and disinhibited eating in children [24], as well as adults [42,75].

### 5.3. Limitations and Future Directions

While this study has several strengths in synthesizing the associations between EF and disinhibited eating patterns across child development, findings should be interpreted in light of limitations. Given the mixed methodology of assessing EF and eating behaviors, it is possible that studies only reported significant findings from their studies that risks publication bias. Second, there is risk of false non-significant associations of inhibitory control and cognitive flexibility, as well as moderating effects of age, gender, or weight status with disinhibited eating due to the small number of studies that met inclusion criteria [76]. The pooling of EF effects on various disinhibited eating behaviors within studies may also have resulted in nonlinear and multifactor effects [77]. Disinhibited eating behaviors while overlapping are also distinct, suggesting associations may vary by individual eating construct [6]. It is also important to acknowledge that while individual factors may be linked to EF and disinhibited eating in youth, child development and behaviors are still largely influenced by the environment [78].

Pediatric weight management intervention often involves self-monitoring of dietary intake, a task that calls on EF processes such as working memory. Future research is needed to examine novel ways to optimize pediatric weight management interventions to account for the potential barrier of lower EF on disinhibited eating and risk for poorer obesity management outcomes. Moving forward, it will be important for researchers and clinicians to account for the complex intersections between EF and eating regulation, both in intervention design and outcome assessment, to optimize pediatric weight management outcomes. It may also be important to educate families on the underlying neurobiology, such as weaker EF processes, that contribute to the maintenance of obesogenic eating patterns and problem-solve ways to support the child or adolescent.

## 6. Conclusions

Taken together, the study findings are consistent with the literature that EF plays a small, yet important, role in disinhibited eating in youth. Although significant effects were not detected for inhibitory control or cognitive flexibility, findings highlight the importance of poorer overall executive function and working memory as risk factors for disinhibited eating across child development. The link between executive dysfunction and disinhibited eating may be a barrier to effective weight management that warrants attention in the field to improve response to pediatric obesity treatment efforts. More research is needed to understand under what genetic, environmental, and individual factors EF affects disinhibited eating behavior and weight management outcomes across child development.

## Figures and Tables

**Table 1:** Descriptive characteristics of included studies in the meta-analysis.

ES	Eating Assessment Method	Eating Assessment(s)	Eating Behavior	EF Assessment Method	EF Assessment(s)	EF Construct(s)	% OW/OB	Gender % Female	Age Range (Mean)	N	Study
0.02	Subjective	DEBQ	EAH EE	Objective	Go/No-Go	IC	N/A	56	7–11 (8.2)	50	[49]
−0.3	Subjective	CEBQ	EAH	Objective	Snack Delay Tower Task Simon Says, Wrapped Gift Task	EF	40	50	3–6 (5.3)	40	[35]
0.05	Objective	Free Access Procedure	EAH	Objective	DOG	IC	50	48.7	7–10 (7.8)	230	[40]
−0.2	Subjective	Child EDE	LOC	Subjective and Objective	BRIEF-GEC Flanker DCCS List Sorting	EF IC CF WM	34.67	58.7	9–12 (10.5)	75	[24]
−0.01	Subjective	CEBQ DEBQ	EAH EE	Objective	Fruit Stroop DOG Cognitive Flexibility Task Digit Span Backwards	IC CF WM	12.9	52.1	6–11 (8.3)	1657	[39]
−0.02	Objective	Free Access Procedure	EAH	Subjective and Objective	CBQ-EC Tapping task DOG Gift Delay Flexible Item Selection	EF IC CF	47.1	47.6	3–5 (4.8)	187	[29]
−0.22	Objective	Ad lib taste test	EAH	Objective	Stop-signal	IC	N/A	64.8	7–9 (8.1)	88	[30]
−0.03	Subjective and Objective	CEBQ Free Access Procedure	EE EAH	Subjective and Objective	DRDP-IC CBQ-EC Flanker Dots Task	EF IC CF	N/A	51.7	4–7 (4.4)	29	[26]
−0.46	Subjective	CEBQ	EE	Subjective	Child Self-Regulation in Eating Questionnaire	EF	N/A	47.5	3–5 (4.2)	254	[27]
0.1	Subjective and Objective	CEBQ Free Access Procedure	EAH	Objective	DOG	IC	47.1	48	4–5 (4.8)	185	[31]
−0.35	Subjective	BES	BE	Subjective	BRIEF-GEC BRIEF-BRI BRIEF-MI	EF IC WM	19.09	58.9	12–18 (14.9)	969	[21]
−0.09	Subjective	ChEDE-Q	BE LOC	Subjective	BRIEF-BRI BRIEF-INH	EF IC	15.5	67.2	10–17 (13.5)	301	[22]
−0.03	Subjective	Ch-EDE	LOC	Subjective	BRIEF-BRI BRIEF-INH	EF IC	10.5	65.3	10–17 (13.5)	133	[25]
−0.2	Objective	Free Access Procedure	EAH	Subjective	CBQ-EC	EF	N/A	52.2	4–6 (4.8)	115	[33]
−0.38	Subjective	EES	EE	Subjective	Self-Control Scale	EF	8.75	56.5	15–18 (16.7)	594	[28]

Note. BE = binge eating; BES = Binge Eating Scale; BRIEF-BRI = Behavioral Inventory of Executive Functions Behavioral Regulation Index; BRIEF-GEC = Brief Rating Inventory of Executive Function Global Executive Composite; BRIEF-Inh = Behavioral Rating Inventory of Executive Functions Inhibition subscale; BRIEF-MI = Behavioral Rating Inventory of Executive Functions Metacognition Index; CBQ-EC = Child Behavior Questionnaire Effortful Control Subscale; CEBQ = Chile Eating and Behavior Questionnaire; CF = cognitive flexibility; Ch-EDE = Child Eating Disorder Examination; ChEDE-Q = Child Eating Disorder Examination Questionnaire; DCCS = dimensional change card sort; DEBQ = Dutch Eating Behavior Questionnaire; DOG = Delay of Gratification; DRDP-IC = Desired Results Developmental Profile Impulsive Control Subscale; EAH = eating in the absence of hunger; EE = emotional eating; EES = Emotional Eating Scale; EF = executive function; ES = mean Fischer Z correlation effect size; IC = inhibitory control; LOC = loss of control; OW/OB = overweight and obese; WM = working memory.

## Data Availability

The datasets generated and/or analyzed during the current study are available from the corresponding author on reasonable request.

## Abbreviations

Executive functioning (EF); eating in the absence of hunger (EAH); emotional eating (EE); loss of control (LOC); and binge eating (BE).
